# Clinical polyneuropathy does not increase with prediabetes or metabolic syndrome in the Japanese general population

**DOI:** 10.1111/jdi.13058

**Published:** 2019-05-14

**Authors:** Seigo Kurisu, Hideyuki Sasaki, Shohei Kishimoto, Kazuhiro Hirayasu, Kinichi Ogawa, Shohei Matsuno, Hiroto Furuta, Mikio Arita, Keigo Naka, Kishio Nanjo, Takashi Akamizu

**Affiliations:** ^1^ Department of Medicine Kihoku Hospital Wakayama Medical University Wakayama Japan; ^2^ First Department of Medicine Wakayama Medical University Wakayama Japan; ^3^ Division of Diabetes and Metabolism Satellite Clinic for Integrative and Anti‐Aging Medicine Wakayama Medical University Wakayama Japan; ^4^ Wakayama Rosai Hospital Wakayama Japan; ^5^ Kansai University of Health Sciences Osaka Japan; ^6^ Health‐promotion Research Center Wakayama Japan

**Keywords:** Metabolic syndrome, Prediabetes, Prevalence of symmetric sensorimotor polyneuropathy

## Abstract

**Aims/Introduction:**

The prevalence of clinical polyneuropathies (ClinPNs) or nerve conduction abnormality (NCA) in the groups stratified by glucose tolerance, individual components of metabolic syndrome (metabolic syndrome [MetS] components: hypertension, dyslipidemia, obesity) and MetS defined by the International Diabetes Federation consensus was investigated in the Japanese general population. Factors associated with ClinPN and NCA were also identified.

**Materials and Methods:**

A total of 625 examinees of regional medical checkup programs were recruited to this cross‐sectional study. ClinPNs were diagnosed by the Toronto Consensus. NCA was judged by at least one bilateral abnormality of sural nerve action potential amplitude or conduction velocity measured by a point‐of‐care nerve conduction device (DPNCheck). Clinical factors associated with ClinPNs or NCA were examined by multiple logistic regression analysis. Deteriorating factors of sural nerve action potential amplitude or conduction velocity values were also investigated in participants without diabetes (*n* = 550).

**Results:**

As for glucose tolerance, ClinPNs or NCA significantly increased only in known diabetes patients compared with other groups. There was no difference between prediabetes and the normal group. The prevalence of ClinPNs and NCA was not significantly related to MetS or MetS’ components, except for frequent NCA in obesity. The factors significantly associated with both NCA and ClinPNs were smoking and known diabetes. In non‐diabetic participants, aging, tall height and hypertension were significant deteriorating factors of nerve conduction functions.

**Conclusions:**

In Japan, ClinPNs and NCA were increased in known diabetes patients, but did not increase in participants with prediabetes, MetS and MetS’ components. Smoking and known diabetes were factors significantly associated with ClinPNs or NCA. Hypertension might be a modifiable deteriorating factor of nerve function.

## Introduction

Diabetic symmetric sensorimotor polyneuropathy (DSPN) has been reported as the most common disorder of chronic distal symmetric polyneuropathies in Western countries^1^. Recently, reports that show a high prevalence of DSPN‐like polyneuropathy in individuals with prediabetes (impaired fasting glucose or impaired glucose tolerance) or metabolic syndrome (MetS) have been increasing^2^, ^3^, ^4^. Thus, neurological screening of patients with prediabetes complaining of symptoms of peripheral neuropathy is recommended by the American Diabetes Association position statement^4^. Most of the studies showing a high prevalence of polyneuropathy (PN) in prediabetes or MetS have been reported from Western countries, where the degree and frequency of obesity is high. There are few reports from East Asia, including Japan.

In the present study, we aimed to examine the difference in prevalence of clinical PNs (ClinPNs) or nerve conduction abnormality (NCA) by the existence of glucose intolerance (prediabetes etc.), MetS and individual constituting factors of MetS (MetS’ components), and to clarify the factors associated with ClinPNs or NCA in a Japanese general population. MetS’ components consisted of hypertension, dyslipidemia and obesity. ClinPNs was diagnosed according to the Toronto Consensus^5^. The nerve conduction study was carried out by using a point‐of‐care nerve conduction device called NC‐stat^®^/DPNCheck™ (DPNCheck, Neurometrix Inc., Waltham, MA, USA). Furthermore, the deteriorating factors of quantitatively assessed nerve functions in a non‐diabetic population were evaluated.

## Methods

### Ethics statement

These protocol and consent procedures were carried out in accordance with the World Medical Association's Helsinki Declaration, and were approved by the ethics board of the Wakayama Medical University (Approval number 92). All participants provided written informed consent.

### Research design and participants

The present cross‐sectional study was designed to elucidate the relationships between ClinPNs or NCA and glucose intolerance, MetS and MetS’ components in a Japanese general population in a rural area. Furthermore, the deteriorating factors to nerve conduction functions (conduction velocity [CV] amplitude of action potential [AMP]) were also assessed in a non‐diabetic population.

We recruited 625 residents (260 men and 365 women, aged 40–75 years) who received regional medical screening programs. The examinees with a positive history for clinical cerebral infarction sequela, renal failure, hypothyroidism or alcoholism were excluded. The participants were stratified into subgroups by the existence of MetS and MetS’ components, such as glucose tolerance, blood pressure, blood lipid levels and obesity.

First, they were stratified into four groups according to glucose tolerance as follows: (i) normal group (*n* = 429, glycated hemoglobin [HbA1c] <6.0% and fasting plasma glucose <100 mg/dL); (ii) newly diagnosed diabetes mellitus group (*n* = 13, HbA1c ≥6.5% and/or fasting plasma glucose ≥126 mg/dL); (iii) known diabetes mellitus group (*n* = 62, who were previously identified as diabetes mellitus); and (iv) prediabetes group (*n* = 120, who did not correspond to normal, newly diagnosed diabetes mellitus, known diabetes mellitus groups). Newly diagnosed diabetes mellitus is considered to have distinct clinical features characterized by a short duration of marked hyperglycemic exposure and less effects of microangiopathies. Recent epidemiological studies have evaluated the prevalence of ClinPNs separately in newly diagnosed diabetes mellitus and known diabetes mellitus^6^, ^7^, ^8^, ^9^, and two of them evaluated only in newly diagnosed diabetes mellitus^7^, ^9^. Thus, we evaluated newly diagnosed diabetes mellitus and known diabetes mellitus separately to compare with previous reports.

The participants were also stratified into three groups by blood pressure (BP; mmHg) according to the guideline for hypertension treatment by the Japan Society of Hypertension 2014 as follows: (i) optimal/normal pressure (O/NBP) group (*n* = 251, BP <130/80 without antihypertensive drug); (ii) elevated BP group (*n* = 77, systolic BP 130–139 and/or diastolic BP 85–89, without antihypertensive drug); and (iii) hypertension (HT) group (*n* = 296, BP ≥140/90, or under antihypertensive treatment). Additionally, the participants were stratified into two groups by blood lipid levels as follows: (i) the normal lipidemia group (*n* = 252, triglyceride >150 mg/dL, low‐density lipoprotein cholesterol >140 mg/dL and high‐density lipoprotein cholesterol <40 mg/dL without lipid lowering drug); and (ii) the dyslipidemia group (*n* = 372, other than normal lipidemia). Furthermore, the participants were stratified into two groups by obesity level as follows: (i) the non‐obese group (*n* = 500, body mass index [BMI] <25); and (ii) the obese group (*n* = 125, BMI ≥25). Finally, they were stratified into two groups, the no MetS group (*n* = 316) and MetS group (*n* = 307). MetS was diagnosed by the International Diabetes Federation consensus worldwide definition of MetS^10^. Lipid‐lowering and antihypertensive drugs were administered in 44% (151/372) of the dyslipidemia group and 69% (205/297) of the HT group.

### Evaluation of neurological functions

Subjective symptoms of PN were determined by asking whether there were any positive symptoms (e.g., “asleep numbness,” prickling or stabbing, burning or aching pain) in the toes, feet or legs by interview. Achilles tendon reflexes (ATR) on both sides were examined in the knee standing position. In order to verify the signs of a symmetric decrease of distal sensation at the lower limbs, quantitative vibration threshold (QVT) at 125 Hz was assessed at both big toe tips using a vibratory sensation meter (AU‐02B™; RION Inc., Tokyo, Japan). The method of QVT measurement as been described previously^11^. Abnormality of QVT was judged by the upper 5th percentile cut‐off value of normal Japanese individuals. As two participants complained of pain in the right toe, QVT at the toes was not examined.

As nerve conduction functions, AMP and CV of the bilateral sural nerves were measured by DPNCheck according to the test manual^12^. Abnormalities of AMP and CV were judged by the lower 5th percentile cut‐off value of normal Japanese individuals. All abnormalities were diagnosed in each participant using regression formulas to calculate the normal limit of QVT, AMP and CV. Details of the regression formulas were described in our previous report^13^. As QVT and AMP depend on age, the cut‐off values were adjusted by age. As CV depends on age and height, the cut‐off value was adjusted by age and height. A total of 12 participants refused the contralateral examination because of a feeling of discomfort while being tested. In these cases, measured values were adopted ad the representative values. The AMP of seven limbs (0.56%) and CV of 12 limbs (0.96%) were undetectable. In these cases, the results were judged as abnormal.

### Diagnosis of ClinPNs and NCA

ClinPNs were diagnosed according to the latest international consensus on diabetic neuropathies by the Toronto diabetic neuropathy expert group (Toronto Consensus)^5^. In the consensus, three characteristic clinical symptoms/signs in the legs for DSPN were proposed as follows: (i) positive neuropathic sensory symptoms; (ii) sign of symmetric decrease of distal sensation; and (iii) unequivocally decreased or absent ATR. Then, DSPN was categorized into four stepwise criteria according to the presence of the above symptoms/signs: (i) “possible DSPN”: one symptom/sign; (ii) “probable DSPN”: two or more symptoms/signs; (iii) “confirmed DSPN”: one or more symptoms/signs with nerve conduction abnormality (small fiber neuropathy [SFN] can be substituted); and (iv) “subclinical DSPN”: nerve conduction abnormality or SFN without any symptoms/signs.

In the present study, bilateral QVT abnormality was substituted as a sign of symmetric decrease of distal sensation in the lower extremities in order to improve accuracy and objectivity. We used three definitions of the Toronto Consensus^5^ as ClinPNs, namely “possible DSPN”, “probable DSPN” and “confirmed DSPN” for analysis. Also, NCA was diagnosed when there was a bilateral abnormality of either AMP or CV, or both.

As small nerve fiber functions were not evaluated in the present study, we could not mention SFN from our data.

### Statistical analysis

First, we assessed the demographic, clinical and nerve function data of the total participants, and their relationship to glucose tolerance. Continuous variable data were analyzed by one‐way anova followed by Fisher's least significant difference method as a post‐hoc test, whereas nominal variables data were analyzed by the χ^2^‐test. Second, we compared the prevalence of markers of polyneuropathy (PN markers; neuropathic symptoms, diminished ATR, abnormal QVT, abnormal AMP, abnormal CV), NCA and ClinPNs between the groups, which were divided by glucose tolerance and MetS’ components. Statistical analyses were made by a χ^2^‐test being followed by a residual analysis as a post‐hoc test. Furthermore, we elucidated the significant associated factors of ClinPNs and NCA by a multiple logistic regression analysis using demographic, habitual factors and MetS’ components as independent variables. The same analysis was also carried out in non‐diabetic participants.

Additionally, the prevalence of prediabetes and newly diagnosed diabetes mellitus in the participants with ClinPNs (“possibly DSPN” or “probable DSPN”, without known diabetes mellitus) of unknown origin was also investigated.

As the influence of MetS’ components might be weaker than overt diabetes, we evaluated the association between actual values of quantitative nerve function parameters (QVT, AMP and CV) and MetS’ components in non‐diabetic participants (*n* = 550). Differences in QVT, AMP and CV of the groups with MetS or MetS’ components and the groups without them were analyzed using anova with Fisher's least significant difference method as a post‐hoc test. Additionally, the significant deteriorating factors to QVT, AMP and CV were analyzed by a forward stepwise regression analysis using demographic, habitual factors and MetS’ components as independent variables. In these analyses, the average values of right and left QVT, AMP, and CV were used for calculation.

Statistical analyses were carried out using statistical software (Statview‐J5.0TM; Hulinks, Tokyo, Japan, and Excel statistics 2010; Social Survey Research Information Co, Ltd, Tokyo, Japan).

## Results

### Characteristics of total participants and the comparison of characteristics among the four groups stratified by glucose tolerance

In total participants, the average BMI and age were 22.6 kg/m^2^ and 62.0 years old; they were a non‐obese slightly elderly population (Table 1). Along with the deterioration of glucose tolerance, age, BMI and waist circumference increased significantly from the level of prediabetes. In the known diabetes mellitus group, the proportion of men and smokers was significantly higher than those in the normal group. Inevitably, fasting plasma glucose and HbA1c significantly increased in the order of normal, prediabetes, newly diagnosed diabetes mellitus and known diabetes mellitus. Systolic blood pressure and triglyceride in the prediabetes, newly diagnosed diabetes mellitus and known diabetes mellitus groups were significantly higher than those in the normal group, and high‐density lipoprotein cholesterol was lower.

**Table 1:** Demographic, clinical and nerve functional data of total participants, and those of four groups (normal, prediabetes, newly diagnosed diabetes and known diabetes) stratified by glucose tolerance

	Total participants		Four groups stratified by glucose tolerance								*P*‐value of the difference
			Normal		Prediabetes		NDM		KDM		
	*n*	M ± SD or *n* (%)	*n*	M ± SD or *n* (%)	*n*	M ± SD or *n* (%)	*n*	M ± SD or *n* (%)	*n*	M ± SD or *n* (%)	
Demographic factors											
Age (years)	625	62.0 ± 9.5	430	60.4 ± 9.9	120	65.2 ± 7.6****	13	66.4 ± 5.6*	62	66.1 ± 6.2****	<0.0001
Sex, Male (%)	260/625 (41.6)		159∇∇/430 (37.0)		57/120 (47.5)		5/13 (38.5)		39∆∆/62 (62.9)		0.0007
Smoking (no, current, previous)	393(63)/50 (8)/181 (29)		284^∆^(66)/37 (9)/108∇∇(25)		73 (60)/6(5)/41 (34)		9 (69)/0 (0)/4 (31)		27∇∇(44)/7 (11)/28∆∆ (45)		0.0097
Alcohol (no, daily, social)	171 (27)/125 (20)/328 (53)		106 (25)/90 (21)/233 (54)		44 (37)/24(20)/52 (43)		5 (33)/1 (8)/7 (54)		16 (26)/10 (16)/36 (58)		0.1362
Height (cm)	625	160.3 ± 8.8	430	160.4 ± 8.4	120	160.1 ± 9.1	13	159.4 ± 11.9	62	160.7 ± 9.8	0.9488
Weight (kg)	625	58.3 ± 10.9	430	57.0 ± 10.2	120	60.9 ± 11.2***	13	63.0 ± 15.0*	62	61.1 ± 12.3**	0.0002
Body mass index (kg/m^2^)	625	22.6 ± 3.3	430	22.1 ± 2.9	120	23.7 ± 3.7****	13	24.8 ± 5.5**	62	23.5 ± 3.5***	<0.0001
Waist circumference (cm)	623	84.2 ± 8.7	428	82.8 ± 8.0	120	87.5 ± 8.3****	13	89.5 ± 14.8**	62	86.9 ± 9.6***	<0.0001
Clinical factors											
Systolic blood pressure (mmHg)	625	128 ± 17	430	126 ± 17	120	132 ± 17***	13	141 ± 15**	62	137 ± 19****	<0.0001
Diastolic blood pressure (mmHg)	625	75 ± 10	430	74 ± 10	120	76 ± 11	13	79 ± 11	62	75 ± 10	0.1771
Fasting plasma glucose (mg/dL)	623	97 ± 17	430	89 ± 6	119	105 ± 7****	13	130 ± 14****####	62	129 ± 31**** ####	<0.0001
Hemoglobin A1c (%)	623	5.6 ± 0.5	430	5.4 ± 0.2	119	5.7 ± 0.3****	13	6.3 ± 0.5****####	61	6.6 ± 0.8**** #### †	<0.0001
Triglyceride (mg/dL)	623	119 ± 70	430	108 ± 57	119	135 ± 73***	13	191 ± 129**** ##	61	147 ± 106****	<0.0001
Total cholesterol (mg/dL)	624	211.5 ± 33.1	430	213 ± 32	120	213 ± 33	13	212 ± 33	61	198 ± 34***##	0.0072
HDL cholesterol (mg/dL)	624	62.6 ± 15.7	430	64 ± 16	120	59 ± 14**	13	63 ± 18	61	56 ± 14***	<0.0001
Nerve functional factors											
Quantitative vibratory thresholds											
Right big toe (dB)	623	18.3 ± 8.4	428	17.5 ± 8.5	120	19.1 ± 7.7	13	21.5 ± 8.8	62	22.1 ± 7.3****#	0.0001
Left big toe (dB)	625	18.5 ± 8.4	430	17.6 ± 8.5	120	19.4 ± 7.3*	13	22.3 ± 8.1*	62	22.4 ± 8.1****#	<0.0001
Amplitude of sural nerve action potential											
Right (μV)	615	14.5 ± 7.7	422	15.2 ± 7.8	120	13.7 ± 7.5	13	11.4 ± 6.6	60	11.7 ± 6.5***	0.0017
Left (μV)	614	13.9 ± 7.4	422	14.7 ± 7.4	119	12.9 ± 7.3*	11	11.5 ± 7.4	62	11.0 ± 6.1***	0.0004
Conduction velocity of sural nerve											
Right (m/s)	613	54.6 ± 4.9	420	55.0 ± 4.7	120	54.2 ± 4.7	13	54.9 ± 4.0	60	52.6 ± 5.9***#	0.0047
Left (m/s)	611	53.7 ± 4.7	422	54.3 ± 4.5	117	53.1 ± 4.5*	11	52.5 ± 4.3	61	50.8 ± 5.2****##	<0.0001

Statistical analyses of continuous variables were carried out by one‐way anova followed by Fisher's least significant difference method as a post‐hoc test. *P < 0.05, **P < 0.01, ***P < 0.001, ****P < 0.0001 versus normal, ^#^ *P* < 0.05, ^##^ *P* < 0.01, ^####^ *P* < 0.0001 versus prediabetes, ^†^ *P* < 0.05 versus newly diagnosed diabetes (NDM). Statistical analyses of nominal variables were carried out using the χ^2^‐test. Then the residual analysis was used as a post‐hoc test. ^∇∇^ *P* < 0.01 (show higher prevalence compared to the other groups), ^∆^ *P* < 0.05 (show lower prevalence compared to the other groups), ^∆∆^ *P* < 0.01 (show higher prevalence compared to the other groups). HDL, high‐density lipoprotein; KDM, known diabetes; M, mean value; SD, standard deviation.

In nerve function data, QVT, AMP and CV of both legs of the known diabetes mellitus group were significantly worse than those of the normal group. Additionally, QVT, AMP and CV of the left leg of the prediabetes group were significantly deteriorated compared with the normal group.

### Prevalence of PN markers, NCA and ClinPNs in each group stratified by glucose tolerance, MetS’ components and MetS

All PN markers except “neuropathic symptoms” and “abnormal QVT” showed a significant tendency to increase in parallel with glucose intolerance (Table 2). However, in comparisons between individual groups, only the known diabetes mellitus group showed a significantly higher prevalence than the other groups. The prevalence of all PN markers in prediabetes was almost equivalent to that in the normal group. The prevalence of “abnormal AMP” and NCA in the newly diagnosed diabetes mellitus group was high, close to the known diabetes mellitus group, but did not reach significant levels because of the small number of patients.

**Table 2:** Prevalence of polyneuropathy markers, nerve conduction abnormality and clinical polyneuropathies in total participants

	Markers of polyneuropathy						Clinical polyneuropathies		
	Neuropathic symptoms	Diminished ATR	Abnormal QVT	Abnormal AMP	Abnormal CV	NCA	Possible DSPN	Probable DSPN	Confirmed DSPN
	*n* (%)	*n* (%)	*n* (%)	*n* (%)	*n* (%)	*n* (%)	*n* (%)	*n* (%)	*n* (%)
Total participants	55/625 (8.8)	68/625 (10.9)	19/624 (3.0)	32/621 (5.1)	35/622 (5.6)	61/624 (9.8)	125/625 (20.0)	16/625 (2.6)	21/624 (3.4)
Glucose tolerance	*P* = 0.201	*P* < 0.001*	*P* = 0.306	*P* < 0.001*	*P* = 0.015*	*P* = 0.001*	*P* < 0.001*	*P* = 0.002*	*P* < 0.001*
Normal	34/430 (7.9)	37/430 (8.6)∇∇	13/429 (3.0)	14/426 (3.3)∇∇	19/429 (4.5)∇∇	31/429 (7.2)∇∇	75/430 (17.4)∇	9/430 (2.09)	9/429 (2.1)∇∇
Pre‐diabetes	10/120 (8.3)	12/120 (10.0)	2/120 (1.7)	7/120 (5.8)	6/120 (5.0)	12/120 (10.0)	23/120 (19.2)	1/120 (0.8)	2/120 (1.7)
NDM	1/13 (7.7)	0/13 (0.0)	0/13 (0.0)	2/13 (15.4)	1/13 (7.7)	3/13 (23.1)	1/13 (7.7)	0/13 (0.0)	0/13 (0.0)
KDM	10/62 (16.1)	19/62 (30.6)∆∆	4/62 (6.5)	9/62 (14.5)∆∆	9/62 (14.5)∆∆	15/62 (24.2)∆∆	26/62 (41.9)∆∆	6/62 (9.7)∆∆	10/62 (16.1)∆∆
Blood pressure	*P* = 0.550	*P* = 0.045*	*P* = 0.964	*P* = 0.768	*P* = 0.069	*P* = 0.190	*P* = 0.105	*P* = 0.451	*P* = 0.540
O/NBP	19/251 (7.6)	20/251 (8.0)	8/250 (3.2)	11/250 (4.4)	9/250 (3.6)	19/251 (7.6)	42/251 (16.7)	5/251 (2.0)	6/251 (2.4)
EBP	6/77 (7.8)	6/77 (7.8)	2/77 (2.6)	4/77 (5.2)	8/77 (10.4)	11/77 (14.3)	13/77 (16.9)	1/77 (1.3)	3/77 (3.9)
HT	30/297 (10.1)	42/297 (14.1)^∆^	9/297 (3.0)	17/294 (5.8)	18/295 (6.1)	31/297 (10.5)	70/297 (23.6)	10/297 (3.4)	12/296 (4.1)
Blood lipid levels	*P* = 0.025*	*P* = 0.888	*P* = 0.756	*P* = 0.438	*P* = 0.082	*P* = 0.136	*P* = 0.261	*P* = 0.427	*P* = 0.807
Normal	30/252 (11.9)	28/252 (11.1)	7/251 (2.8)	15/250 (6.1)	19/250 (7.6)	30/251 (12.0)	56/252 (22.2)	8/252 (3.2)	9/251 (3.6)
Dyslipidemia	25/372 (6.7)	40/372 (10.7)	12/372 (3.2)	17/370 (4.6)	16/371 (4.3)	31/372 (8.3)	69/372 (18.5)	8/372 (2.2)	12/372 (3.2)
Obesity level	*P* = 0.107	*P* = 0.018*	*P* = 0.102	*P* < 0.001*	*P* = 0.992	*P* = 0.020*	*P* = 0.803	*P* = 0.447	*P* = 0.310
Non‐obese	49/500 (9.8)	47/500 (9.4)	18/499 (3.6)	17/496 (3.4)	28/498 (5.6)	42/500 (8.4)	99/500 (19.8)	14/500 (2.8)	15/500 (3.0)
Obese	6/125 (4.8)	21/125 (16.8)	1/125 (0.8)	15/125 (12.0)	7/124 (5.6)	19/124 (15.3)	26/125 (20.8)	2/125 (1.6)	6/124 (4.8)
Metabolic syndrome (IDF)	*P* = 0.977	*P* = 0.029*	*P* = 0.043*	*P* = 0.057	*P* = 0.263	*P* = 0.789	*P* = 0.280	*P* = 0.654	*P* = 0.883
No	28/316 (8.9)	26/316 (8.2)	14/316 (4.4)	11/314 (3.5)	21/315 (5.6)	30/316 (9.5)	58/316 (18.4)	9/316 (2.8)	11/316 (3.5)
Yes	27/307 (8.8)	42/307 (13.7)	5/306 (1.6)	21/305 (6.9)	14/3.5 (4.6)	31/306 (10.1)	67/307 (21.8)	7/307 (2.3)	10/306 (3.3)

Prevalence of the same items in the groups that were stratified by glucose tolerance, metabolic syndrome are and individual components of metabolic syndrome are also shown. *Statistically significant *P*‐value. Statistical analyses of nominal variables were carried out using the χ^2^‐test. Then, the residual analysis was used as a post‐hoc test. ^∇^ *P* < 0.05 (show lower prevalence compared to the other groups), ^∇∇^ *P* < 0.01 (show higher prevalence compared to the other groups), ^∆^ *P* < 0.05 (show higher prevalence compared to the other groups), ^∆∆^ *P* < 0.01 (show higher prevalence compared to the other groups). ATR, Achilles tendon reflex; AMP, amplitude of sensory nerve action potential of sural nerve; CV, conduction velocity of sural nerve; DSPN, diabetic symmetric polyneuropathy; EBP, elevated blood pressure; HT, hypertension; KDM, known diabetes; NDM, newly diagnosed diabetes; O/NBP, optimal/normal blood pressure; QVT, quantitative vibratory perception threshold.

As for blood pressure, only “diminished ATR” in the HT group increased significantly among the indicators of PN. As for dyslipidemia, the prevalence of “neuropathic symptoms” in the dyslipidemia group was significantly lower compared with the normal group. As for obesity, “diminished ATR”, “abnormal AMP” and NCA increased significantly. As for MetS, “diminished ATR” and “abnormal QVT’ in MetS (yes) group increased and decreased significantly compared to MetS (no) group, respectively.

### Associated factors with NCA and ClinPNs by multiple logistic regression analysis

The results of multiple logistic regression analyses assessing associated factors with NCA and ClinPN (“probable DSPN”, “confirmed DSPN”) in total and non‐diabetic participants are shown on the left and right side of Table 3. In these analyses, we used demographic, habitual (smoking and alcohol drinking) factors and MetS’ components as independent variables, and NCA, “probable DSPN” and “confirmed DSPN” as dependent variables.

**Table 3:** Associated factors with nerve conduction abnormality and clinical polyneuropathies in total and non‐diabetic participants by using multiple logistic regression analysis

Dependent variable	Total participants						Non‐diabetic participants					
	NCA		Probable DSPN		Confirmed DSPN		NCA		Probable DSPN		Confirmed DSPN	
	*P* = 0.0008* (*n* = 620)		*P* = 0.0243* (*n* = 621)		*P* = 0.0085* (*n* = 620)		*P* = 0.2855 (*n* = 546)		*P* = 0.0590 (*n* = 547)		*P* = 0.5603 (*n* = 546)	
Independent variable	OR (95% CI)	*P*	OR (95% CI)	*P*	OR (95% CI)	*P*	OR (95% CI)	*P*	OR (95% CI)	*P*	OR (95% CI)	*P*
Age	1.00 (0.96–1.04)	0.988	1.14 (1.03–1.27)*	0.013*	1.03 (0.96–1.10)	0.432	1.00 (0.96–1.04)	0.967	1.19 (1.03–1.38)	0.017	1.05 (0.96–1.14)	0.313
Sex (male)	0.97 (0.31–3.01)	0.960	0.23 (0.02–2.26)	0.209	0.32 (0.04–2.75)	0.302	1.03 (0.29–3.66)	0.959	0.06 (0.00–1.37)	0.078	0.27 (0.02–3.87)	0.313
Height	1.03 (0.97–1.09)	0.336	1.06 (0.93–1.20)	0.388	1.11 (1.00–1.24)	0.053	1.02 (0.96–1.09)	0.555	1.01 (0.86–1.20)	0.873	1.07 (0.94–1.23)	0.307
Weight	0.99 (0.82–1.06)	0.717	1.02 (0.87–1.19)	0.819	0.96 (0.84–1.09)	0.499	0.97 (0.89–1.05)	0.477	1.09 (0.90–1.32)	0.368	0.95 (0.81–1.13)	0.579
Waist circumference	1.02 (0.95–1.10)	0.532	0.96 (0.83–1.11)	0.621	1.02 (0.90–1.15)	0.791	1.05 (0.97–1.14)	0.261	0.97 (0.81–1.15)	0.702	1.06 (0.90–1.24)	0.511
Glucose tolerance												
Prediabetes	1.47 (0.69–3.12)	0.318	0.34 (0.04–2.92)	0.325	0.75 (0.15–3.74)	0.726	1.30 (0.61–2.76)	0.502	0.21 (0.02–2.01)	0.176	0.68 (0.14–3.39)	0.634
NDM	3.97 (0.91–17.4)	0.067	0.00 (0.00–)	0.998	0.00 (0.00–)	0.998						
KDM	3.65 (1.68–7.93)*	0.001*	4.06 (1.22–13.5)*	0.022*	8.95 (2.95–27.2)*	0.001*						
Blood pressure												
EBP	1.46 (0.62–3.44)	0.382	0.54 (0.06–5.33)	0.601	1.18 (0.25–5.58)	0.834	1.69 (0.67–4.23)	0.266	0.00 (0.00‐)	0.997	1.45 (0.24–8.88)	0.685
HT	1.01 (0.55–2.22)	0.789	1.31 (0.37–4.61)	0.672	1.42 (0.43–4.74)	0.567	1.06 (0.48–2.34)	0.879	1.58 (0.35–7.12)	0.553	0.86 (0.19–3.78)	0.839
Dyslipidemia	0.53 (0.30–0.96)*	0.038*	0.45 (0.14–1.44)	0.178	0.67 (0.24–1.86)	0.443	0.68 (0.34–1.34)	0.266	1.03 (0.21–5.00)	0.972	1.61 (0.40–6.38)	0.501
Smoking												
Previous	2.23 (0.74–6.80)	0.156	7.38 (1.11–48.9)*	0.038*	2.00 (0.26–15.6)	0.509	2.48 (0.74–8.32)	0.140	22.3 (1.58–312)	0.021	3.55 (0.33–38.2)	0.296
Current	2.39 (1.01–5.71)*	0.049*	2.06 (0.32–13.3)	0.124	2.67 (0.50–14.3)	0.252	2.18 (0.84–5.66)	0.110	1.29 (0.10–17.2)	0.850	0.87 (0.09–8.22)	0.906
Alcohol												
Social	0.82 (0.32–2.07)	0.674	3.83 (0.59–24.9)	0.160	0.42 (0.07–2.47)	0.339	0.64 (0.23–1.80)	0.394	2.48 (0.17–35.5)	0.504	0.00 (0.00‐)	0.998
Daily	1.48 (0.73–2.99)	0.275	3.28 (0.57–18.8)	0.183	1.23 (0.39–3.95)	0.723	0.96 (0.42–2.16)	0.912	3.17 (0.28–36.1)	0.353	0.45 (0.10–8.22)	0.906

*Statistically significant values. Statistical analyses were carried out using multiple logistic regression analysis. AMP, amplitude of sensory nerve action potential of sural nerve; CI, confidence interval; CV, conduction velocity of sural nerve; DSPN, diabetic symmetric polyneuropathy; EBP, elevated blood pressure; HT, hypertension; KDM, known diabetes; NDM, newly diagnosed diabetes; OR, odds ratio.

Significant factors associated with NCA were known diabetes mellitus (odds ratio [OR] 3.65, 95% confidence interval [CI] 1.68–7.93) and current smoking (OR 2.39, 95% CI 1.01–5.71), whereas dyslipidemia (OR 0.53, 95% CI 0.30–0.96) was a protective factor. Significant associated factors with “probable DSPN” were aging (OR 1.14, 95% CI 1.03–1.27), known diabetes mellitus (OR 4.06, 95% CI 1.22–13.5) and previous smoking (OR 7.38, 95% CI 1.11–48.9). Significant associated factor with “confirmed DSPN” was only known diabetes mellitus (OR 8.95, 95% CI 2.95–27.2).

No significant associated factor was detected in non‐diabetic participants. A possible reason for this finding might be related to the low prevalence of NCA (7.9%: 43/546), “probable DSPN” (1.8%: 10/547) and “Confirmed DSPN” (2.0%: 11/547).

### Prevalence of prediabetes or newly diagnosed diabetes mellitus in the participants with ClinPNs (“possible DSPN” or “probable DSPN”) of unknown origin

Analysis was carried out of the participants (*n* = 563) excluding the known diabetes mellitus group. The prevalence of prediabetes in the group with and without “possible DSPN” was 23.2% (23/99) and 20.9% (97/464), respectively. The prevalence of prediabetes in the group with and without “probable DSPN” was 10.0% (1/10) and 21.5% (119/553), respectively. There was no significant increase of prediabetes in unknown ClinPNs participants (χ^2^ = 0.26, 0.78; *P* = 0.61, 0.38).

In the same way, the prevalence of newly diagnosed diabetes mellitus in the group with and without “possible DSPN” was 1.0% (1/99) and 2.6% (12/464), and the prevalence of newly diagnosed diabetes mellitus in the group with and without “probable DSPN” was 0% (0/10) and 2.4% (13/553), respectively. Thus, newly diagnosed diabetes mellitus also did not increase in unknown ClinPNs (χ^2^ = 0.89, 0.78; *P* = 0.24, 0.62).

### Relationships between actual values of quantitative nerve function parameters and MetS’ components in non‐diabetic participants (*n* = 550)

Measurements of QVT, AMP and CV in the groups with and without MetS or MetS’ components are shown in Table 4. The QVT and AMP of the prediabetes group were significantly worse than those of the normal group. The QVT, AMP and CV of the HT group were also significantly worse as compared with the O/NBP group. In addition, the AMP of the obese group, and the AMP and CV of the MetS group were significantly lower than those of the other groups.

**Table 4:** Relationships between quantitative nerve functions and the components of metabolic syndrome in Japanese non‐diabetic participants

(a) Influence of metabolic syndrome and its related factors on QVT, AMP and CV						
	QVT (dB)		AMP (mV)		CV (m/s)	
	*n*	M ± SD	*n*	M ± SD	*n*	M ± SD
Glucose tolerance	*P* = 0.043		*P* = 0.026		*P* = 0.126	
Normal	422	17.6 ± 8.1	427	14.9 ± 7.0	427	54.5 ± 4.9
Pre‐diabetes	120	19.2 ± 7.1	120	13.3 ± 6.8	120	53.7 ± 4.2
Blood pressure	*P* < 0.001		*P* < 0.001		*P* = 0.001	
O/NBP	237	16.5 ± 7.9	236	16.0 ± 7.4	236	55.1 ± 4.3
EBP	69	16.6 ± 7.7	69	15.8 ± 7.0	69	53.3 ± 3.8**
HT	243	19.7 ± 7.7***$$	242	12.7 ± 6.1*** ^$$$^	242	53.8 ± 5.3**
Blood lipid levels	*P* = 0.227		*P* = 0.613		*P* = 0.960	
Normal	228	17.6 ± 8.0	225	14.7 ± 7.2	228	54.3 ± 4.8
Dyslipidemia	321	18.3 ± 7.8	319	14.4 ± 6.9	319	54.3 ± 4.8
Obesity level	*P* = 0.540		*P* < 0.001		*P* = 0.955	
Non‐obese	449	18.0 ± 7.9	447	15.1 ± 7.1	447	54.3 ± 4.7
Obese	100	17.5 ± 7.9	100	12.1 ± 6.1	100	54.3 ± 4.9
Metabolic syndrome (IDF)	*P* = 0.371		*P* < 0.001		*P* < 0.001	
No	282	17.7 ± 8.0	281	15.8 ± 7.4	281	53.6 ± 4.6
Yes	265	18.3 ± 7.6	264	13.2 ± 6.3	264	55.0 ± 4.8

Statistical analyses were carried out by anova followed by Fisher's least significant difference method as a post‐hoc test. **P < 0.01, ***P < 0.001 versus the optimal/normal blood pressure (O/NBP) group, ^$$^ *P* < 0.01, ^$$$^ *P* < 0.01, versus elevated blood pressure (EBP) group. The values were indicated as average of left and right. HT, hypertension; QVT, quantitative vibratory perception threshold; AMP, amplitude of sensory nerve action potential; CV, conduction velocity of sural nerve.

Table 5 shows the results of stepwise regression analysis using QVT, AMP and CV as dependent variables, and demographic factors, MetS components and habitual factors as independent variables. QVT showed significant positive correlations with age, height and alcohol intake. AMP showed significant negative correlations with age, height, bodyweight and hypertension, and positive correlations with sex (male) and dyslipidemia. Furthermore, CV showed a significant negative correlation with age, sex (male), height and hypertension, and positive correlations with bodyweight.

**Table 5:** Association between demographic/clinical factors and QVT, AMP and CV (stepwise regression analysis)

Dependent variables	QVT (dB)	AMP (mV)	CV (m/s)
*n*	546	544	544
*R* ^2^	0.358	0.188	0.261
*P* value	<0.0001	<0.0001	<0.0001

Independent variables	Standard RC	*P*‐value	Standard RC	*P*‐value	Standard RC	*P*‐value
Age (years)	0.618	<0.0001	−0.353	<0.0001	−0.216	<0.0017
Sex (female: 0, male: 1)	NA		0.373	<0.0001	−0.204	0.0003
Height (cm)	0.282	<0.0001	−0.147	0.0226	−0.414	<0.0001
Weight (kg)	NA		−0.261	<0.0001	0.154	0.0038
Waist circumference (cm)	NA		NA		NA	
Glucose tolerance Normal: 0, prediabetes: 1	NA		NA		NA	
Blood pressure O/NBP: 0, EBP: 1, HT: 2	NA		−0.126	0.0038	−0.090	0.0254
Blood lipid level Normal: 0, dyslipidemia: 1	NA		0.097	0.0170	NA	
Smoking No: 0, previous: 1, current: 2	NA		NA		NA	
Alcohol No: 0, social: 1, daily: 2	0.085	<0.0001	NA		NA	

Statistical analyses were carried out by forward stepwise multiple regression analysis. Glucose tolerance, blood pressure and lipid abnormalities were categorized into dummy variables and evaluated. AMP, amplitude of sensory nerve action potential; CV, conduction velocity; NA, not adopted; QVT, quantitative vibratory perception threshold; RC, regression coefficient.

## Discussion

One of our aims was to investigate the difference in prevalence of NCA and ClinPNs (assessed by large fiber function tests) depending on the presence or absence of glucose intolerance, MetS and MetS’ components, and to evaluate the clinically associated factors with NCA and ClinPNs. As results, two major findings were obtained.

First, as for glucose intolerance, the prevalence of NCA or ClinPNs (“possible, probable and confirmed DSPN”) in prediabetes was almost equivalent to that of the normal group. Only in the known diabetes mellitus group did NCA and ClinPNs show a significantly higher prevalence compared with the other groups. From these findings, in the Japanese population, it was suggested that clinically evident symptoms/signs of PN might not appear unless hyperglycemia corresponding to diabetes persists to some extent.

Several epidemiological reports from Western countries have shown an increase of the prevalence of PN in prediabetes. Ziegler *et al*.^2^ reported a significantly higher prevalence of PN determined by a Michigan Neuropathy Screening Instrument examination score >2^14^ in the impaired glucose tolerance group (13%) compared with the normal group (7%), and there was a significant association between waist circumference or peripheral artery disease and PN in the general population of Germany. Similarly, a higher prevalence of PN (vibratory and/or touch sensation impairment in feet diagnosed with 10‐g monofilament and 64‐Hz tuning fork) in the prediabetes group (24%) compared with the normal group (11%) was reported in an elderly population in Germany^6^. In a 3‐year prospective study of a high‐risk group of MetS or diabetes patients in the USA, the final prevalence of PN (Michigan Neuropathy Screening Instrument examination score >2) in the prediabetes and newly diagnosed diabetes mellitus groups was 49% and 50%, respectively, which was significantly higher than that in the normal group (29%)^7^. The authors also reported that impaired glucose tolerance was a significant risk factor for PN^7^. Meanwhile, in a Chinese community‐based population, the prevalence of PN (diagnosed by a combination of neuropathic symptoms, ATR, vibratory sensation due to tuning fork and so on) in the normal, prediabetes and newly diagnosed diabetes mellitus groups was 1.5%, 2.8% and 8.4%, respectively. A significant increase in PN prevalence could not be observed in the prediabetes group^8^.

According to a report from Dyck *et al*.^9^, the prevalence of strictly diagnosed PN based on a nerve conduction study in multiple nerves in the normal, prediabetes and newly diagnosed diabetes mellitus groups was 2, 1.7 and 7.8%, respectively. Though, PN prevalence in the prediabetes group did not change, that in the newly diagnosed diabetes mellitus group significantly increased. The present data also showed a high prevalence of “abnormal AMP” and NCA in newly diagnosed diabetes mellitus group close to those in the known diabetes mellitus group, whereas the high prevalence in the newly diagnosed diabetes mellitus group did not reach a significant level due to the small number (Table 2). Therefore, it might be thought that NCA emerges from the early stages of diabetes.

Because the diagnostic method of PN is different, it is difficult to compare the prevalence between these reports. Although the reason why the prevalence of ClinPNs in the prediabetes group did not increase similarly to reports from Western countries is unknown, the lower average values of BMI (22.6 kg/m^2^) and height (160 cm) of the present participants compared with those of Western individuals’ BMI (27–30 kg/m^2^) and height (165–170 cm) might influence the difference in PN prevalence.

All these epidemiological studies evaluated only large fiber function. Many studies showing small fiber structural and functional abnormalities^15^, ^16^, ^17^ in prediabetes and MetS have accumulated. Thus, further detailed epidemiological studies are required to elucidate the prevalence of symptomatic SFN in the general population.

Second, MetS and MetS’ components (hypertension, dyslipidemia and obesity) had little effect on the prevalence of NCA or ClinPNs. Multiple logistic regression analyses showed that the clinically significant associated factors with NCA and ClinPNs (“probable or confirmed DSPN”) were smoking and known diabetes mellitus. It is controversial whether an association between PN and MetS is significant or not in a population without diagnosed diabetes. Callaghan *et al*.^3^ reported that the prevalence of PN increased in parallel with the number of MetS’ components in all the groups of normal, prediabetes, and newly diagnosed diabetes mellitus in a cohort study of elderly participants. In contrast, Lee *et al*.^7^ reported that MetS was not independently associated with PN (diagnosed by Michigan Neuropathy Screening Instrument scores or vibration thresholds). Several reports^18^, ^19^, including meta‐analysis, have reported that smoking is a related factor of PN in diabetes patients. In the present study, smoking was a significant associated factor with NCA or “probable DSPN” in the total participants. Therefore, smoking and diabetes might increase the risk of PN additively.

In the present study, no significant association was found between aging and NCA and “confirmed DPN”. The reason might be related to the fact that AMP and CV abnormalities were determined by age‐adjusted criteria. In contrast, dyslipidemia showed favorable effects on NCA and AMP values. The reason for these findings is unknown. The relatively well‐controlled average lipid levels (triglyceride: 142 mg/dL, total cholesterol: 221 mg/dL, high‐density lipoprotein cholesterol: 59 mg/dL) in the dyslipidemia group by sufficient use of lipid‐lowering drugs (44% of dyslipidemia) might be related to the result.

From another viewpoint on the association between MetS and PN, reports that showed a high prevalence of MetS’ components (prediabetes, newly diagnosed diabetes mellitus, obesity, dyslipidemia etc.) in the patients with symptomatic PN of unknown origin have accumulated from Western countries. Singleton *et al*.^20^ reported a high prevalence of prediabetes (34%: 36/107) and newly diagnosed diabetes mellitus (12%: 13/107) in patients with idiopathic symptomatic PN. Smith AG *et al*.^21^ reported that the prevalence of MetS’ components in 219 patients with idiopathic PN was higher than the general population, especially dyslipidemia, which was remarkably higher. Visser *et al*.^22^ reported that the prevalence of MetS in 249 idiopathic PN patients was significantly higher than that in the control group (54% vs 34%). In addition, hypertension and abdominal obesity were significantly more prevalent in idiopathic PN patients than in the control group by multivariate analysis. In the same way, we also investigated the prevalence of early glucose intolerance (prediabetes and newly diagnosed diabetes mellitus) in individuals with “possible DSPN” or “probable DSPN” of unknown origin. As a result, the prevalence of prediabetes or newly diagnosed diabetes mellitus did not increase in the participants with an unknown “possible DSPN” or “probable DSPN” compared with the participants without PN. These findings might suggest that the prevalence of early glucose tolerance in the ClinPNs patients does not increase in the Japanese population. However, a larger study using a greater number of symptomatic PN patients would be necessary to confirm the hypothesis, because the number of the participants with ClinPNs of unknown origin in the present study was too small.

Another aim of this study was to clarify the clinical deteriorating factors of quantitatively evaluated nerve function (QVT, AMP and CV) in a non‐diabetic population, because it was confirmed that diabetes was strongly associated with PN. Although a significant difference in QVT and AMP by anova between the groups with and without prediabetes was observed, the significance disappeared in multivariate stepwise regression methods. Several previous studies^3^, ^9^, ^23^ have also reported that nerve conduction parameters by a routine method in prediabetes did not significantly deteriorate compared with the normal participants. Therefore, prediabetes seems not to be a significant deteriorating factor for nerve conduction function. As for blood pressure, significant associations between hypertension and the deterioration of AMP and CV were shown by the both of uni‐ and multivariate analyses. A significant association between hypertension and PN has been reported in non‐diabetic individuals^22^ and also in diabetes patients^18^. Thus, the present finding might suggest that hypertension could be a possible deteriorating factor to nerve conduction function also in Japanese non‐diabetic individuals.

Furthermore, multivariate stepwise regression analysis showed that height was significantly associated with the deterioration of peripheral nerve function (QVT, AMP and CV) in the lower legs. A significant decreasing effect of taller height on nerve conduction was also shown in the previous report^24^ in normal Caucasian participants. In contrast, the same analysis also showed that bodyweight correlated negatively to AMP and positively to CV, interestingly. This finding might be physiologically explained to be the result of thick subcutaneous tissue of the lower legs. Namely, the thicker subcutaneous tissue might cause more attenuation of AMP, and the higher heat retention might facilitate CV.

An advantage of the present study was that PN was assessed by a nerve conduction test with an objective and quantitative method, and that the several types of ClinPNs of the Toronto Consensus were investigated. Age‐adjusted evaluation of QVT and AMP, and age/height evaluation of CV might also be a merit of our study. Although, the sural nerve conduction study was not carried out by conventional methods, the high accuracy and reliability of the point‐of‐care nerve conduction device type nerve conduction device (DPNCheck) for identification of DSPN have been reported^25^, ^26^. Andersen *et al*.^27^ diagnosed the “confirmed DSPN” of Toronto Consensus in type 2 diabetes patients using DPNCheck, and showed a significant association between higher HbA1c levels/slopes of HbA1c trajectories and DSPN.

The major limitation of the present study was that small nerve fiber functions were not evaluated. Another limitation is that as the present data were obtained from a cross‐sectional study, targeting the inhabitants of a rural area, there is a possibility that the results might be different in the residents of urban areas.

Summarizing all our investigations, we might conclude on the relationships between PN and glucose intolerance or MetS in the Japanese general population as follows. First, although clinically evident PN does not occur unless diabetes persists to some extent, NCA might occur earlier in diabetes. Second, the significant factors associated with NCA and ClinPNs are smoking and known diabetes mellitus, whereas MetS or MetS’ components are not associated with NCA and ClinPNs. Third, hypertension might be a modifiable possible deteriorating factor of nerve conduction function within the normal range. Thus, the prevalence and related factors of PN between Western and Japanese people might be not identical. In order to elucidate the reason, a large‐scale longitudinal study using the same PN evaluation methods including quantitative examinations of both Western and Japanese populations will be necessary.

## Disclosure

The authors declare no conflict of interest.
